# The Side Effects of Facial Implants

**DOI:** 10.1155/2016/1910567

**Published:** 2016-04-26

**Authors:** Adela Ioana Uta, Claudia Elena Uta, Lia Anguelova Valentinova, Carlos Isanta Pomar

**Affiliations:** ^1^Primary Care Center San Jose, Zaragoza, Spain; ^2^Primary Care Center La Vall d'Uixó 1, Calle Santuario de Cabañas sn, La Vall d'Uixó, 50013 Zaragoza, Spain; ^3^National Institute of Endocrinology Parhon, Bucharest, Romania; ^4^The University of Zaragoza, Zaragoza, Spain

## Abstract

This case report is about a 64-year-old woman who presented at the Emergency Walk-in Center with palpebral edema as well as various erythematous plaques in supraciliary and malar areas that have been gradually worsening a couple of days prior to presentation. While talking about history, the patient mentioned she was attending, for about four months, an Esthetic Private Clinic, where she was injected in various sessions with Metacrill®, as a facial lift, for beauty purpose. Due to suspecting an allergic reaction to the Metacrill and the failure of the initial treatments, she was referred to the dermatologist. After failed attempts to treat the patient with corticosteroids and antibiotics, the patient was sent for autoimmunity consultation at the hospital where she received an immunosuppressive treatment with Tacrolimus and was not presenting new symptoms ever since.

## 1. Clinical Case

**Figure 1:**
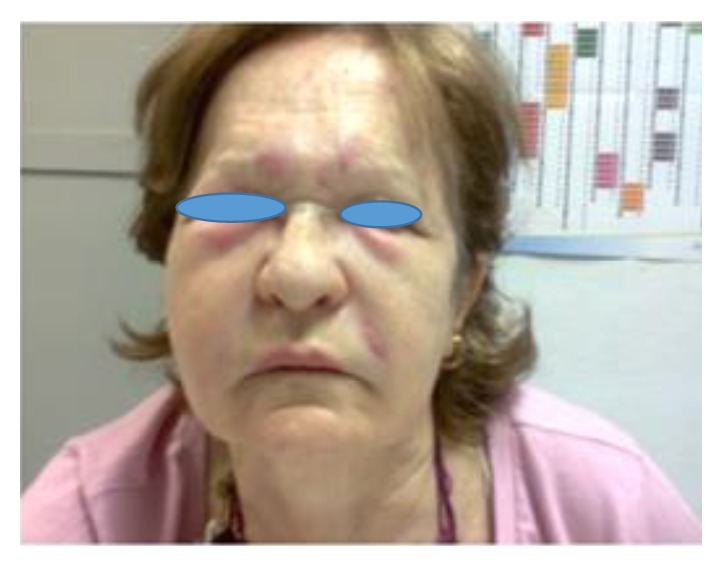
First visit to the Emergency Room. Erythematous plaques in supraciliary and malar areas.

A 64-year-old woman presents to the Emergency Room with palpebral edema as well as various erythematous plaques in supraciliary and malar areas that appeared and have gradually worsened a couple of days prior to presentation (Figure 1). Initially the patient did not share having changed her daily facial hygiene, making any diet changes, getting any animal bites, or taking any new medication.

On exanimation, the patient had facial swelling, pain on palpation, and flushing.

A full blood count was performed with no alterations of the blood formula, neither leucocytes nor the eosinophils, and no inflammatory markers or IgE elevated.

Still suspecting an allergic reaction, she was given treatment with corticosteroids and antihistaminic medication, both intramuscularly and with a unique dose.

Four days later, the patient returned to the Primary Care consultation due to recurrence of her symptoms and was referred once again to the Emergency Room where she was clinically diagnosed with cellulitis and received antibiotic treatment (amoxicillin with clavulanic acid) along with corticosteroids and antihistaminic medication once again.

**Figure 2:**
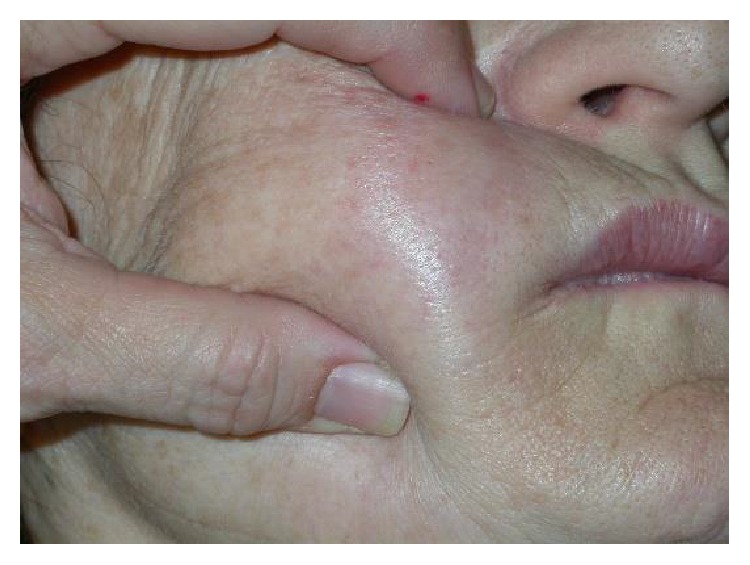
Granuloma 3*∗*3 cm located in supraciliary area, right side.

Although she referred a short period of improvement of the symptoms, the swelling and rash reappeared when the treatment was suspended along with another two nodules of hard consistency localized on the cheekbone of the right facial side (Figure 2).

During the fourth Primary Care consultation, the patient was interrogated more thoroughly and only then she mentioned that she was visiting, for about four months, an Esthetic Private Clinic, where she was injected in various sessions with Metacrill, as a facial lift, for beauty purpose. Apparently no other patient suffered the same symptoms.

Due to suspecting an allergic reaction to the Metacrill and the failure of the initial treatments, she was referred to the dermatologist.

**Figure 3:**
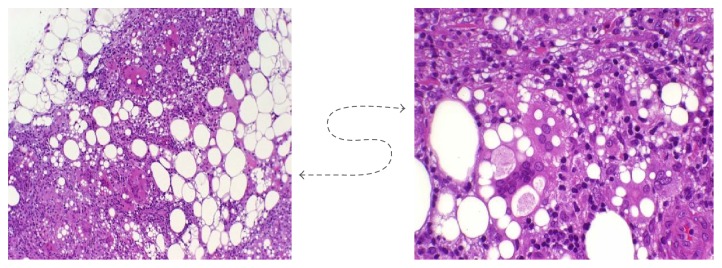
Chronic Edematous Panniculitis, histopathologic finding. Cellular reaction, with formation of fibrosis, and no giant cells.

During the dermatology consultation, a biopsy of one of the granulomas was performed, being diagnosed as Chronic Edematous Panniculitis to a foreign body and probably caused by a polymeric material in the context of an iatrogenic infiltration of esthetic filler (Figure 3).

The patient received a new treatment with corticosteroids, in a decreasing dose associated with antibiotics (doxycycline orally).

When trying to lower the dose of corticoid, the patient suffers a new episode of edema and as a consequence the dose of oral corticoid is raised and is associated with a corticoid topic cream.

**Figure 4:**
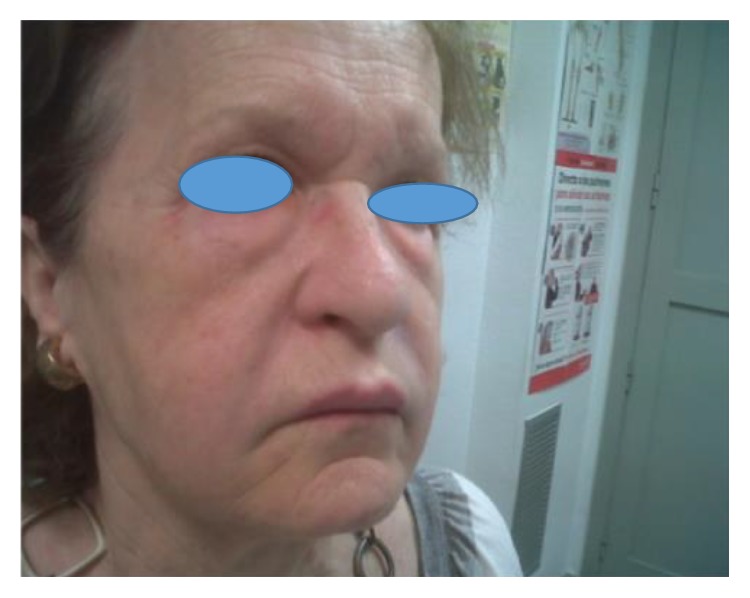
After the immunosuppressive treatment, the patient presented a regression of the granulomas.

Due to the bad evolution, the patient is sent to the autoimmunity consultation at the hospital where she receives an immunosuppressive treatment with Tacrolimus and was not presenting new symptoms ever since. Every three months, the levels of the immune response are being tested to avoid toxicity (Figure 4).

## 2. Conclusions

The Metacrill implant is used for treating frontal wrinkles, outlines and lips fillers, back of the hands, facial outline, nasolabial groove, dorsum, and tip of the nose. It is also used for correction of the cellulitis and facial lipodystrophy in HIV positive patients [1].

The side effects of Metacrill with a smaller impact are the erythema, redness at the site of injection, the hematomas especially around periorbital area that can be avoided by applying ice, whitening of the most superficial skin surface that disappears spontaneously, and a discreet tumefaction of the treated wrinkles.

The more severe side effects are hyperpigmentation of the injection area, probably by neovascularization, abscess of the injection area that disappears in a few months, facial edema and an allergic reaction of the injected material, persistent erythema with antibodies for the implant, granulomas, nodules or local fibrous indurations, small calcifications or necrosis of the skin, or mucous membrane of the lips [2, 3].

The treatment for any of side effects of Metacrill usually is symptomatic. In case of side effect with a smaller impact like erythema, the treatment is only local with hygienic measures, applying ice without needing pharmacological therapy.

The granulomas, the persistent erythema, or nodules are being treated with corticoid decreasing course and only in the most severe cases when not responding to the usual treatment the immunosuppressive therapy is indicated [4, 5].

*What Is Already Known about This Topic?* Metacrill has been used in medical implants since 1936. The published cases of complications are described as a result of technical errors during the application, when the implants touch surface planes of epidermis [6].

*What Does This Study Add?* Currently, the beauty industry is thriving and the exposure of population to products and procedures for a better appearance has become more often, which means a higher risk to be exposed to the side effects [6].

## 3. Bottom Line

Soft tissue augmentation is a process of implanting tissues or materials where needed to restore a youthful or more aesthetic look to the face, more and more patients look for aesthetic improvement through less invasive procedures than before, and the demand for injectable soft tissue fillers to treat wrinkles in the body or to augment soft tissues has grown dramatically.

Although injectable filler substances are replacing conventional tissue transfer and the number of biocompatible fillers continues to increase, rapid absorption and overall disappointing long-term outcomes are common drawbacks for nearly all natural fillers, which are available at present.
